# Increased medial posterior tibial slope is associated with medial compartment osteoarthritis of the knee

**DOI:** 10.1002/jeo2.70676

**Published:** 2026-02-26

**Authors:** Moses K. D. El Kayali, Luis V. Bürck, Clemens Gwinner, Stephan Oehme, Alan Getgood, Lorenz Pichler

**Affiliations:** ^1^ Center for Musculoskeletal Surgery Charité – Universitätsmedizin Berlin Berlin Germany; ^2^ Julius Wolff Institute Berlin Institute of Health at Charité – Universitätsmedizin Berlin Berlin Germany; ^3^ ASPETAR, Orthopaedic and Sports Medicine Hospital Doha Qatar; ^4^ Department of Orthopedics and Trauma‐Surgery Medical University of Vienna Vienna Austria

**Keywords:** medial compartment osteoarthritis, medial posterior tibial slope, PTS, tibiofemoral biomechanics

## Abstract

**Purpose:**

The primary aim was to assess the association between medial posterior tibial slope (MPTS) and medial knee osteoarthritis (OA), hypothesising higher MPTS values in OA knees. The secondary aim was to evaluate the prevalence of increased MPTS (≥ 12°), hypothesising a higher prevalence in the OA group compared with the non‐OA group.

**Methods:**

Patients were retrospectively identified from an institutional database. The OA group comprised patients with isolated medial knee OA (Kellgren–Lawrence [K/L] grade ≥3), while a non‐OA group (K/L grade <1) served as controls. Patients with prior knee surgery, fracture, tumour, or radiographs unsuitable for MPTS measurement were excluded. Groups were frequency‐matched for age, sex, and body mass index (BMI). MPTS and medial proximal tibial angle (MPTA) were measured by two raters. Between‐group comparisons, multivariable logistic regression, and receiver operating characteristic (ROC) analysis were performed (*p* < 0.05).

**Results:**

A total of 562 patients (304 OA, 258 non‐OA) were included. Groups did not differ in age (59.8 ± 7.7 vs. 59.6 ± 5.1 years), sex (50.0% vs. 56.6% female), BMI (28.5 ± 3.7 vs. 27.9 ± 3.5 kg/m²), or MPTA (86.6° ± 3.6° vs. 87.2° ± 3.4°) (all *p* > 0.050). Mean MPTS was higher in the OA group (7.8° ± 3.5° vs. 6.6° ± 2.4°; *p* < 0.001), and MPTS ≥ 12° was more frequent in OA knees (13.2% vs. 1.9%; *p* < 0.001). Each 1° increase in MPTS was associated with higher odds of medial OA (OR 1.14, confidence interval [CI] 1.07–1.20), while MPTS ≥ 12° increased the odds of medial OA 7.9‐fold (OR 7.9, CI 3.07–20.49). ROC analysis showed modest discriminative ability for medial OA (AUC = 0.59; optimal cutoff, 8.6°).

**Conclusion:**

Increased MPTS was significantly associated with medial knee OA. These findings highlight sagittal tibial geometry as a potentially relevant morphological factor and support further longitudinal investigation.

**Level of Evidence:**

Level III, diagnostic study.

**Abbreviations**
ACL: anterior cruciate ligament
AUC: area under the curve
BMI: body mass index
CI: confidence interval
ICC: intra‐class correlation coefficient
K/L: Kellgren–Lawrence grade
MPTS: medial posterior tibial slope
OA: osteoarthritis
OR: odds ratio
PTS: posterior tibial slope
ROC: receiver operating characteristic
UKA: unicompartmental knee arthroplasty

## INTRODUCTION

The posterior tibial slope (PTS) is a recognised risk factor for anterior cruciate ligament (ACL) injury and ACL graft failure in both adolescents and adults [14, 18, 29, 35, 40]. An increased PTS leads to greater anterior tibial translation, resulting in increased strain on the native ACL or reconstructed ACL graft [16]. Importantly, the PTS represents a modifiable anatomical factor associated with ACL graft failure, and slope‐decreasing osteotomies have demonstrated favourable outcomes in the setting of ACL revision surgery [2, 12, 24].

Beyond its influence on ligament stability, the PTS substantially affects tibiofemoral joint biomechanics. An increased PTS shifts the location of peak contact pressure posteriorly and elevates overall joint contact forces [1, 8, 11, 16, 21, 34, 36, 37]. As contact pressure is a key determinant of tissue damage [3, 4], it is biomechanically plausible that an increased PTS contributes to cartilage degeneration and the development of tibiofemoral osteoarthritis (OA) over time. However, clinical evidence linking increased PTS to knee joint degeneration remains limited [22, 26], and the potential association between PTS and medial compartment OA has not been clearly established.

The primary aim of this study was to investigate the association between PTS and medial compartment OA of the knee joint. It was hypothesised that patients with medial compartment OA (Kellgren–Lawrence grade [K/L] [25] ≥3) would exhibit significantly higher PTS values than patients without OA (K/L grade < 1) The secondary aim was to assess the prevalence of increased PTS (≥12°), with the hypothesis that such values would be more frequently observed in the OA group compared to the non‐OA group.

## MATERIAL AND METHODS

This retrospective comparative study was approved by the local institutional ethics committee (Charité–Universitätsmedizin Berlin) University (Nr. EA2/016/21) and conducted in accordance with the principles of the Declaration of Helsinki.

### Patients

Patients were identified from an institutional radiographic database comprising individuals who underwent standardised knee radiographs at a single academic orthopaedic surgery center between February 2013 and October 2022 for clinical diagnostic or preoperative assessment. Based on radiographic findings, two groups were formed: a medial compartment OA group and a non‐OA control group. The OA group included patients aged 18 years or older who underwent primary, isolated medial unicompartmental knee arthroplasty (UKA) for isolated medial compartment OA of the knee joint with K/L grade ≥3 [25]. Patients with radiographic OA of the lateral or patellofemoral compartments (K/L grade ≥1), prior ipsilateral knee surgery, or combined procedures were excluded.

Frequency matching for age, body mass index (BMI), and sex was performed to minimise potential confounding. The medial OA cohort served as the reference population. From the institutional imaging database, patients aged 18 years or older without radiographic OA (K/L grade <1 in all knee compartments) were selected using stratified random sampling based on sex and predefined BMI strata. Age strata were defined in 5‐year intervals, and BMI strata in 5 kg/m^2^ intervals. This approach ensured comparable distributions of demographic and anthropometric variables between groups. Exclusion criteria for both groups included a history of knee fracture, tumour, prior ipsilateral knee surgery (including ligamentous, bony, or soft‐tissue procedures, e.g., meniscectomy), metabolic bone disorders, or incomplete patient records. Radiographs were further excluded if they did not permit accurate PTS assessment due to insufficient tibial shaft length (<15 cm visible), malrotation or tilt greater than 5 mm as measured on the distal femoral condyles, low image resolution, or non‐digital film quality [10, 15, 38]. Demographic data collected included age, sex, and BMI which were obtained from the patients' electronic medical records.

### Radiographs

Lateral, weight‐bearing radiographs of the affected knee were obtained either during outpatient clinic visits or at the time of surgical indication. Radiographs were acquired using a standardised protocol: the knee was positioned with the detector aligned parallel to the sagittal plane and the central X‐ray beam directed at the patellofemoral joint line. All images were verified for correct femoral condyle overlap to ensure standardised lateral alignment before measurement. All images were calibrated using a standard reference marker of 25.4 mm (1 inch). Radiographs were obtained using a digital radiography system (XGEO GC85A, Samsung, Seoul, South Korea).

### Measurement technique

**Figure 1:**
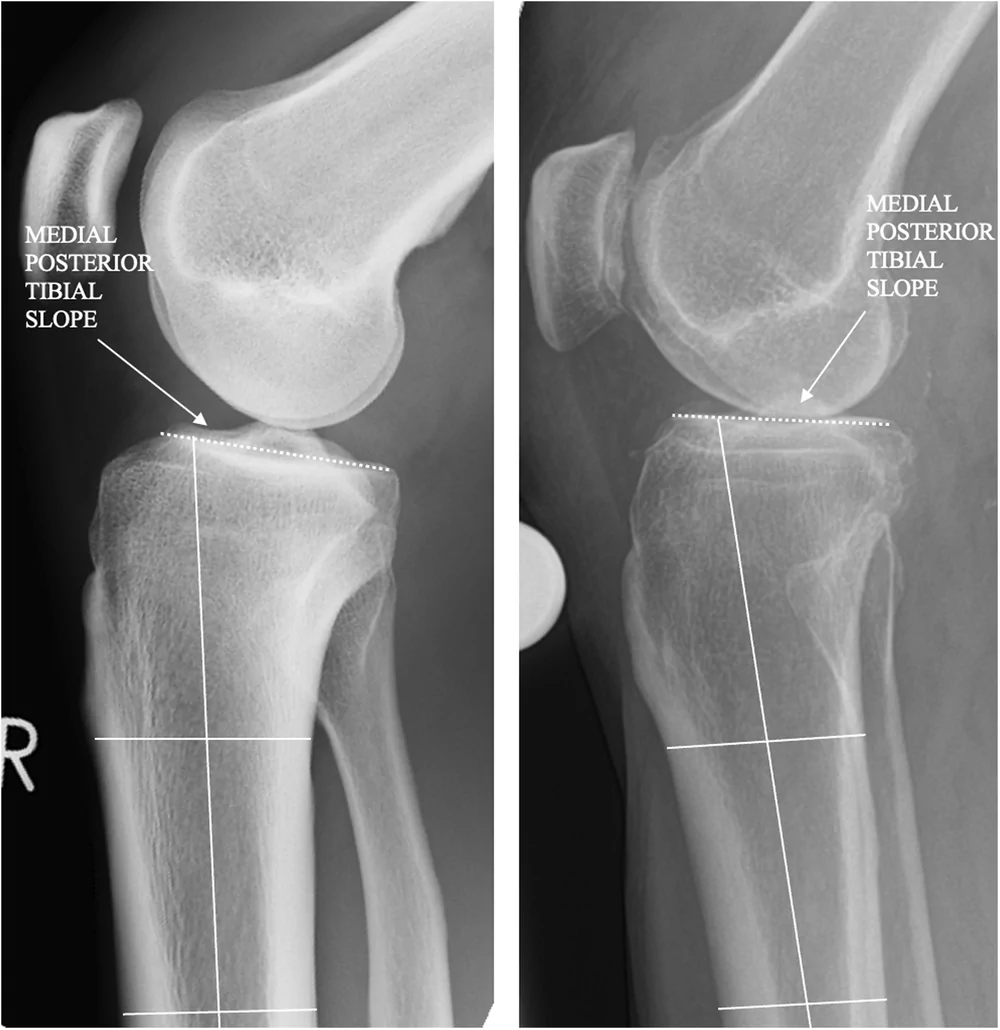
Measurement of MPTS. Lateral radiographs of a non‐OA knee (left) and a knee with medial compartment osteoarthritis (right) showcasing measurement of medial posterior tibial slope. MPTS, medial posterior tibial slope; OA, osteoarthritis.

K/L grading was used to classify OA severity on anterior‐to‐posterior, weightbearing radiographs based on standard radiographic criteria [25]. Measurement of the PTS was performed on both patient groups on standardised lateral knee radiographs according to the method described by Dejour and Bonnin [13], using the medial tibial plateau as the reference point, as recommended by a recent expert consensus statement [38]. Accordingly, this measurement is referred to as the medial posterior tibial slope (MPTS) throughout the manuscript. To determine the proximal tibial diaphyseal axis, a line was drawn between two points identified at equal distances from the anterior and posterior cortices: one just below the tibial tubercle and another located 10 cm distally. At the level of the tibiofemoral joint, a reference line was drawn perpendicular to this axis. The posterior inclination of the tibial plateau was then measured by drawing a line between the most superior points of the anterior and posterior rims of the medial tibial plateau. The angle formed between this line and the perpendicular reference line was defined as the MPTS (Figure 1). MPTS measurements were recorded in degrees and rounded to one decimal place. To control for potential coronal alignment differences between groups, the medial proximal tibial angle (MPTA) was additionally measured on anterior‐to‐posterior, weightbearing radiographs according to the method described by Petersen and Engh [31].

All measurements were performed independently by two observers experienced in musculoskeletal imaging, who were blinded to clinical data, using a PACS workstation (Centricity RIS‐I 4.2 Plus, GE Healthcare, Chicago, IL, USA). Inter‐ and intra‐rater reliability was assessed by calculating the intraclass correlation coefficient (ICC), which was categorised as follows: slight (0–0.2), fair (0.21–0.4), moderate (0.41–0.6), good (0.61–0.8), or excellent (>0.8) [28]. For intra‐rater reliability, both observers repeated all measurements on a randomly selected subset of 50 radiographs after a minimum period of 4 weeks, blinded to their initial measurements. Inter‐ and intra‐rater reliability were excellent for all measurements (ICC > 0.8).

### Statistics

All extracted data were compiled and summarised using Microsoft Excel (version 16.78, Microsoft Corporation, Redmond, WA, USA). Statistical analysis was performed using IBM SPSS Statistics (version 28.0, IBM Corp., Armonk, NY, USA).

To estimate the statistical power achieved by the final sample, a post hoc power analysis was performed in G*Power (Version 3.1.9.6) for a two‐tailed independent‐samples *t*‐test (*α* = 0.05). Using the observed effect size (Cohen's *d* = 0.38) derived from the MPTS means and standard deviations, the achieved power was 0.99.

Continuous variables are reported as means, standard deviations, ranges, and 95% confidence intervals (CI), and categorical variables as counts and percentages. The Shapiro–Wilk test was used to assess normality, and Levene's test was applied to evaluate the homogeneity of variances.

The Welch *t*‐test was used to compare the MPTS between the medial OA and non‐OA groups, accounting for unequal variances. The proportion of cases with a MPTS ≥ 12° and categorical variables were compared using the chi‐square (*χ*²) test. This cut‐off was selected because a MPTS of ≥12° is commonly cited in the literature as a clinically relevant value when considering slope‐decreasing osteotomy in the context of ACL revision surgery [38]. A two‐sided *p* < 0.050 was considered statistically significant.

To assess the independent association between MPTS and the presence of medial compartment OA, binary logistic regression analyses were performed with OA status (OA = 1, non‐OA = 0) as the dependent variable and MPTS as the independent variable. The model was adjusted for age, sex, BMI, and MPTA. The results were reported as odds ratios (OR) with corresponding CI. A secondary model dichotomised MPTS at a threshold of ≥12° to evaluate the categorical association with OA. Further, a receiver operating characteristic (ROC) curve analysis was conducted to assess the discriminative ability of MPTS for identifying medial compartment OA. The area under the curve (AUC) was calculated, and the optimal cutoff value was determined using Youden's *J* statistic, identifying the point maximising sensitivity and specificity.

## RESULTS

**Figure 2:**
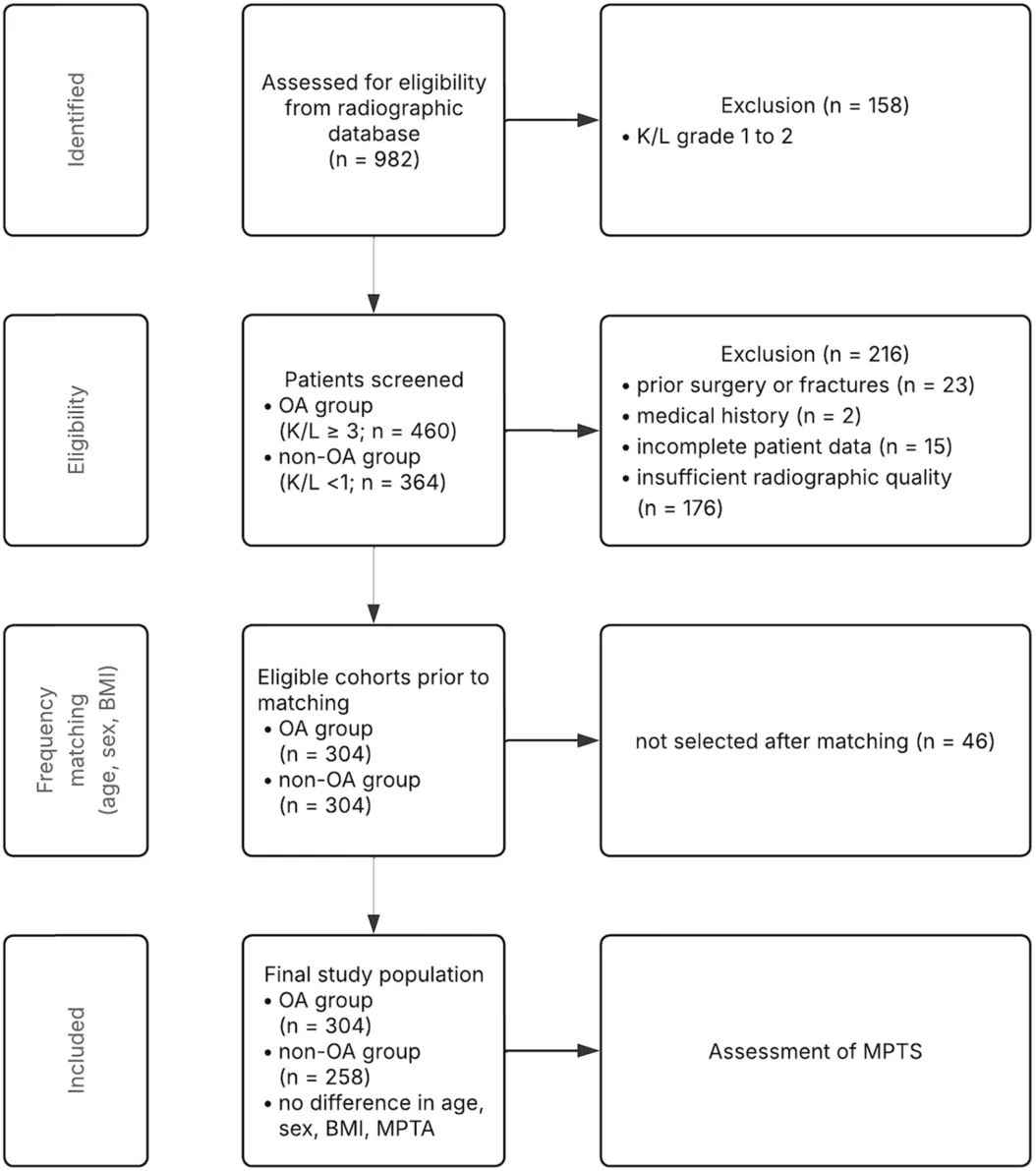
Flow chart of patient selection. BMI, body mass index; K/L, Kellgren/Lawrence grade; MPTA, medial proximal tibial angle; MPTS, medial posterior tibial slope; OA, osteoarthritis.

A total of 562 patients (304 OA, 258 non‐OA; mean age, 59.7 years; 53.0% female; mean BMI, 28.2 kg/m^2^) were included in the analysis (Figure 2). No significant differences in age, sex, BMI, or MPTA were observed between groups (*p* > 0.050) (Table 1).

**Table 1:** Patients characteristics.

Variable	Medial OA (*n* = 304)	Non‐OA (*n* = 258)	*p*‐value
Age, years	59.8 ± 7.7	59.6 ± 5.1	0.640^a^
Sex, female, *n* (%)	152 (50.0)	146 (56.6)	0.580^b^
BMI, kg/m^2^	28.5 ± 3.7	27.94 ± 3.5	0.070^a^
MPTA (°)	86.6 ± 3.6	87.2 ± 3.4	0.060^a^

Abbreviations: BMI, body mass index; MPTA, medial proximal tibial angle; OA, osteoarthritis.

^a^ Welch *t*‐test;

^b^ Chi‐square test. Values represent means and standard deviations if not stated otherwise.

The MPTS was 7.8° ± 3.5° (CI, 7.4°–8.1°; range, 0.6°–17.9°) in the OA group and 6.6° ± 2.4° (CI, 6.3°–6.9°; range, 0.3°–13.2°) in the control group. The mean difference between groups was 1.2° ± 0.3° (CI, 0.7°–1.7°), representing statistically significantly higher MPTS among OA patients (*p* < 0.001, Welch *t*‐test).

**Figure 3:**
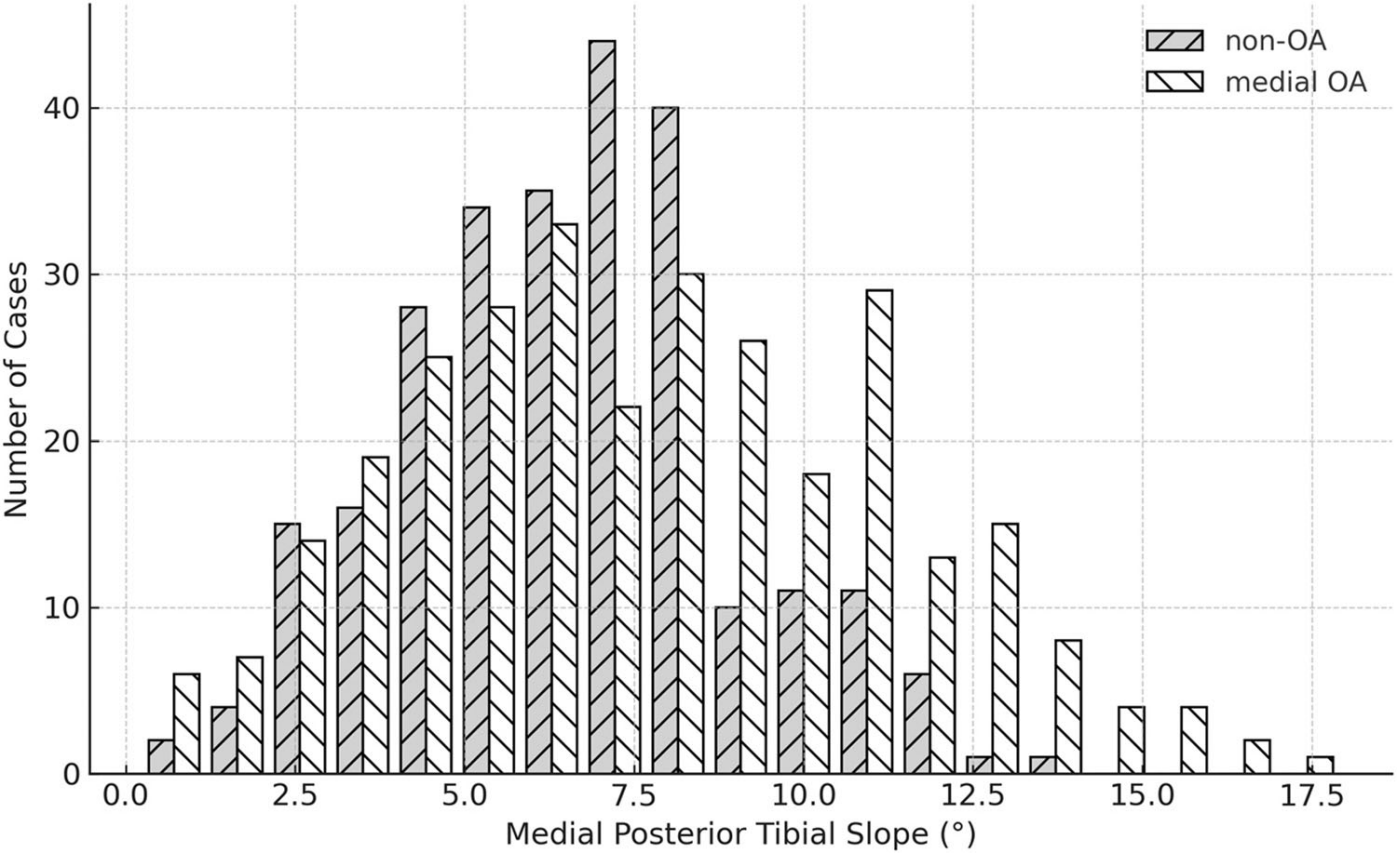
Distribution of MPTS in OA knees versus non‐OA knees. Distribution of MPTS values in patients with OA and non‐OA knees. Bars indicate the number of cases within each MPTS range. The medial OA group demonstrates a right‐shifted distribution, indicating generally higher MPTS values compared with the non‐OA group. MPTS, medial posterior tibial slope; OA, osteoarthritis.

A MPTS of ≥12° was observed in 13.2% (*n* = 40) of the OA group and 1.9% (*n* = 5) of controls (*p* < 0.001, *χ*² test), reflecting a right‐shift toward higher MPTS values seen in the medial OA group (Figure 3).

In the multivariable logistic regression model adjusted for age, sex BMI, and MPTA, each 1° increase in MPTS was associated with a 14% increase in the odds of medial OA (OR 1.14, 95% CI 1.07–1.20; *p* < 0.001). A MPTS of ≥12° increased the odds of medial OA by 7.9‐fold (OR 7.9, 95% CI 3.07–20.49; *p* < 0.001).

**Figure 4:**
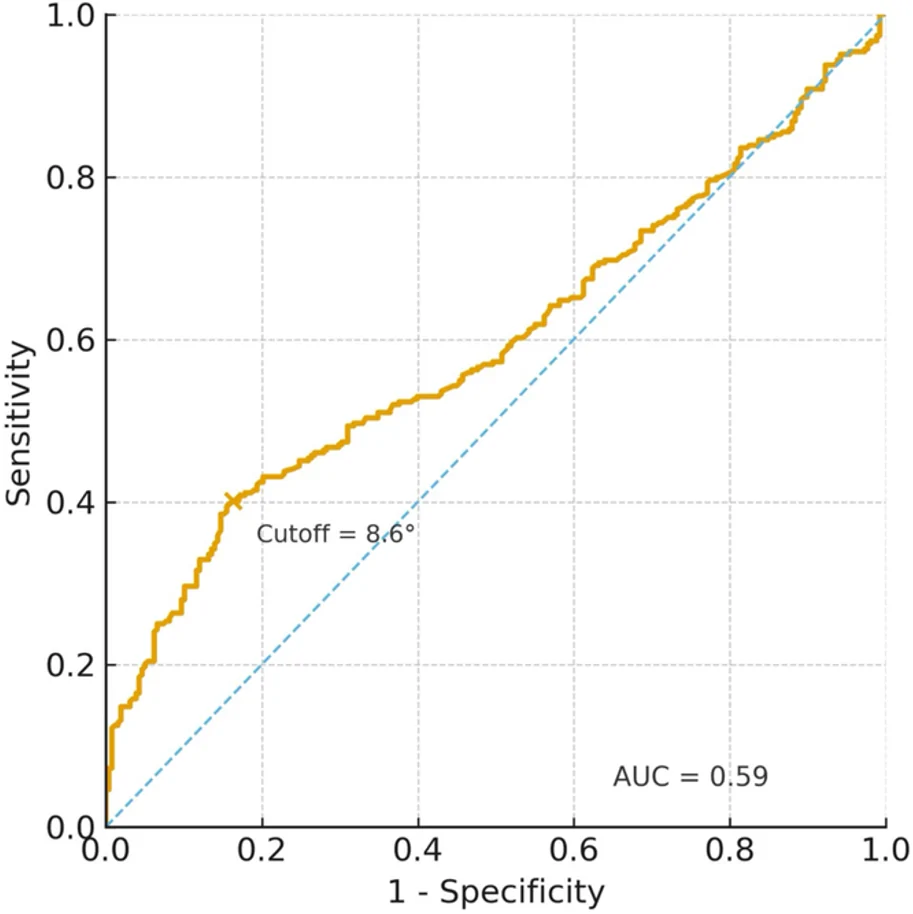
ROC curve for MPTS discriminating medial OA from non‐OA knees. The area under the curve was 0.59, indicating a modest discriminative ability. The optimal MPTS cutoff determined by Youden's J statistic was 8.6°, corresponding to 39.5% sensitivity and 84.4% specificity for identifying medial compartment OA. The diagonal reference line represents no discriminative performance. MPTS, medial posterior tibial slope; OA, osteoarthritis; ROC, receiver operating characteristic.

ROC curve analysis demonstrated a modest discriminative ability of MPTS for identifying medial compartment OA (AUC = 0.59). The optimal cutoff determined by Youden's *J* statistic was 8.6°, corresponding to 39.5% sensitivity and 84.4% specificity (Figure 4).

## DISCUSSION

The key finding of this study was that patients with medial compartment OA demonstrated significantly higher MPTS values compared with patients without radiographic OA (7.8° ± 3.5° vs. 6.6° ± 2.4°). This supports an association between increased MPTS and medial compartment OA, with a higher proportion of MPTS of ≥12° observed among affected patients (13.2% vs. 1.9%). These findings suggest that an increased MPTS may represent a previously underrecognized morphological feature associated with degenerative changes of the medial tibiofemoral joint.

While the PTS is well established as a determinant of knee joint biomechanics, the sagittal plane has received comparatively little attention in the context of cartilage degeneration and OA. Biomechanical studies have shown that increasing the PTS induces an anterior shift of tibial position, thereby increasing load on the native or reconstructed ACL [16, 36]. Clinically, this relationship has major implications as PTS represents a recognised risk factor for ACL injury, as well as ACL graft failure, and assessment of the PTS has become an essential part of the diagnostic workup in those patient groups [9, 35, 38]. Consequently, slope‐decreasing osteotomies are increasingly performed to restore physiological sagittal alignment and mitigate abnormal shear forces in ACL revision surgery, demonstrating that sagittal plane alignment and associated biomechanical effects can be surgically modified [1, 2, 12].

From a broader biomechanical perspective, an increased PTS translates the femoral condyles posteriorly, shifting the location of peak pressure toward the posterior tibial plateau and increasing the magnitude of joint contact forces [1, 11]. It is well established that abnormal joint loading contributes to cartilage matrix breakdown and OA over time [7, 32, 33]. Given the pronounced effect of PTS on tibiofemoral load distribution, it is plausible that an increased PTS similarly promotes degenerative changes. While most established anatomical risk factors of knee joint OA focus on the coronal plane [27], the findings of this study indicate that sagittal‐plane geometry, particularly the MPTS, may also play an important role in tibiofemoral load imbalance. This biomechanical rationale is supported by clinical evidence, as previous studies have demonstrated associations between increased PTS and cartilage damage in the patellofemoral and in the tibiofemoral compartments [22, 26].

Notably, beyond the difference in mean PTS values, the data revealed a right‐shifted distribution of MPTS and a markedly higher frequency of MPTS ≥ 12° in the OA group. This distributional pattern parallels the findings of Weiler et al. [39], who compared ACL‐injured with ACL‐intact knees and found that, although mean PTS values did not differ substantially, the ACL‐injured cohort showed an accumulation of outlier slopes, reflecting a shift toward higher values. Such distributional trends across distant joint pathologies suggest that increased PTS may represent a shared morphological predisposition contributing to altered joint mechanics and joint degeneration.

Although the present results demonstrate only an association rather than a causal relationship, these findings suggest that sagittal tibial geometry represents a relevant morphological factor in the pathogenesis of medial compartment OA through abnormal load distribution [21]. As slope‐decreasing osteotomies are applied effectively in ACL revision surgery to mitigate the biomechanical consequences of an increased PTS, similar principles may warrant future investigation for the management of early medial compartment degeneration. Likewise, high tibial osteotomies performed for varus malalignment and early medial OA have shown favourable outcomes [5, 17, 20]. Building on these concepts, combined biplanar osteotomies addressing both coronal and sagittal deformities could potentially restore a more physiological load distribution and slow disease progression in selected patients with excessive PTS, coronal malalignment, and early unicompartmental OA. This strategy is already applied in the ACL revision setting with concomitant coronal deformity [19, 23, 30]. Prospective biomechanical and clinical studies are required to verify these assumptions and define appropriate indications.

## LIMITATIONS

This study has several limitations that should be acknowledged. First, its retrospective design precludes any inference of causality, and the observed association between increased MPTS and medial compartment OA cannot determine which is the initiating factor. It remains unclear whether an increased PTS predisposes the knee to degenerative changes or whether progressive osteoarthritic remodelling secondarily alters tibial morphology and thereby increases the PTS. However, the large sample size and the absence of significant differences between groups in age, sex, BMI, and MPTA reduce the likelihood that the observed association is solely attributable to confounding. Second, OA severity was classified using radiographic K/L grading rather than magnetic resonance imaging‐based assessment of cartilage status, and only patients with advanced medial OA scheduled for UKA were included. Thus, these findings may not fully represent early or pre‐radiographic stages of joint degeneration. Furthermore, this analysis focused exclusively on the medial compartment, and only the MPTS was measured. However, prior studies suggest that the lateral PTS and the asymmetry between medial and lateral PTS can also influence risk for ACL graft failure [6]. Prospective longitudinal studies integrating biomechanical and imaging assessments are warranted to clarify whether increased PTS contributes causally to the onset or progression knee OA.

## CONCLUSION

An increased MPTS was significantly associated with medial compartment knee OA. Higher MPTS values were associated with increased odds of medial OA, and MTPS ≥ 12° were more prevalent among affected patients. These findings highlight the sagittal tibial geometry as a potentially relevant morphological factor associated with medial compartment degeneration and support further longitudinal investigation to clarify causal relationships.

## AUTHOR CONTRIBUTIONS

Each named author has substantially contributed to conducting the underlying research and drafting this manuscript.

## CONFLICT OF INTEREST STATEMENT

Alan Getgood: Consultant for Smith and Nephew, Stock ownership in Personalised Surgery and Kyniska Robotics.

## ETHICS STATEMENT

Please include the name of the institutional review board (IRB) and the approval number. If not applicable, please state so. The study protocol was approved by the local ethics committee ((EA2/016/21)), and the study was conducted in accordance with the Declaration of Helsinki. Written informed consent was obtained from all patients included in this study.

## Data Availability

The data sets generated and analysed during the current study are available from the corresponding author upon reasonable request.
